# Validation of the diagnostic criteria for IgG4-related kidney disease (IgG4-RKD) 2011, and proposal of a new 2020 version

**DOI:** 10.1007/s10157-020-01993-7

**Published:** 2021-01-04

**Authors:** Takako Saeki, Mitsuhiro Kawano, Tasuku Nagasawa, Yoshifumi Ubara, Yoshinori Taniguchi, Motoko Yanagita, Shinichi Nishi, Michio Nagata, Satoshi Hisano, Yutaka Yamaguchi, Hideki Nomura, Takao Saito, Hitoshi Nakashima

**Affiliations:** 1 Department of Internal Medicine, Nagaoka Red Cross Hospital, Senshu 2-297-1, Nagaoka, Niigata 940-2085 Japan; 2 Department of Rheumatology, Graduate School of Medical Science, Kanazawa University, 13-1 Takara-machi, Kanazawa, Ishikawa 920-8641 Japan; 3 Division of Nephrology, Endocrinology, and Vascular Medicine, Tohoku Univesity Hospital, 1-1 Seiryo-machi, Aoba-ku, Sendai, Miyagi 980-8574 Japan; 4 Rheumatology Department and Okinaka Memorial Institute for Medical Research, Toranomon Hospital, 2-2-2 Toranomon, Minato-ku, Tokyo, 105-8470 Japan; 5 Department of Endocrinology, Metabolism, Nephrology and Rheumatology, Kochi Medical School Hospital, Kochi University, 185-1 Kohasu, Oko-cho, Nankoku-shi, Kochi 783-8505 Japan; 6 Department of Nephrology, Graduate School of Medicine, Kyoto University, 54 Shogoin Kawahara-cho, Sakyo-ku, Kyoto, Kyoto 606-8507 Japan; 7 Division of Nephrology and Kidney Center, Kobe University Graduate School of Medicine, 7-5-2, Kusunoki-cho, Chuo-ku, Kobe, Hyogo 650-0017 Japan; 8 Department of Pathology, Graduate School of Comprehensive Human Sciences, University of Tsukuba, 1-1-1 Tennodai, Tsukuba, Ibaraki 305-8575 Japan; 9 Department of Pathology, University of Occupational and Environmental Health, 1-1, Iseigaoka, Yahatanishi-ku, Kitakyushu-shi, Fukuoka 807-8555 Japan; 10 Yamaguchi’s Pathology Laboratory, Chiba, Japan; 11 Department of General Medicine, Kanazawa University Hospital, 13-1 Takara-machi, Kanazawa, Ishikawa 920-8641 Japan; 12 Sanko Clinic, Fukuoka, 4-9-3 Roppon-Matsu, Chuo-ku, Fukuoka, 810-0044 Japan; 13 Division of Nephrology and Rheumatology, Department of Internal Medicine, Faculty of Medicine, Fukuoka University, 7-45-1 Nanakuma, Jonan-ku, Fukuoka, 814-0180 Japan

**Keywords:** IgG4-related disease, IgG4-related kidney disease, Diagnostic criteria

## Abstract

**Background:**

In 2011, the IgG4-related kidney disease (IgG4-RKD) working group of the Japanese Society of Nephrology proposed diagnostic criteria for IgG4-RKD. The aim of the present study was to validate those criteria and develop a revised version.

**Methods:**

Between April 2012 and May 2019, we retrospectively collected Japanese patients with kidney disease, for whom data on serum IgG4 values and/or immunohistological staining for IgG4 in renal biopsy samples were available. These patients were classified as IgG4-RKD or non-IgG4-RKD based on the diagnostic criteria for IgG4-RKD 2011, and the results were evaluated by expert opinion. Accordingly, we developed some revised versions of the criteria, and the version showing the best performance in the present cohort was proposed as the IgG4-RKD criteria for 2020.

**Results:**

Of 105 included patients, the expert panel diagnosed 55 as having true IgG4-RKD and 50 as mimickers. The diagnostic criteria for IgG4-RKD 2011 had a sensitivity of 72.7% and a specificity of 90.0% in this cohort. Of the 15 patients with true IgG4-RKD who were classified as non-IgG4-RKD, all lacked biopsy-proven extra-renal lesions, although many had clinical findings highly suggestive of IgG4-RD. The revised version to which “bilateral lacrimal, submandibular or parotid swelling, imaging findings compatible with type 1 autoimmune pancreatitis or retroperitoneal fibrosis” was added as an item pertaining to extra-renal organ(s) improved the sensitivity to 90.9% while the specificity remained at 90.0%.

**Conclusion:**

The revised version has considerably improved test performance after addition of the new extra-renal organ item (imaging and clinical findings).

## Introduction

IgG4-related disease (IgG4-RD) is a fibroinflammatory condition characterized by infiltration of abundant IgG4-positive plasma cells with fibrosis, and usually an elevated serum IgG4 concentration [1, 2]. In 2011, the Japanese IgG4 team, organized by the Ministry of Health, Labour, and Welfare (MHLW) of Japan, published comprehensive diagnostic (CD) criteria for IgG4-RD [3]. Organ-specific diagnostic criteria for IgG4-RD have also been published by specialist Japanese medical societies, as IgG4-RD can affect various organs and the diagnostic tools employed therefore differ accordingly [4]. At present in Japan, the MHLW recommends combined use of the CD criteria and organ-specific criteria for the diagnosis of IgG4-RD. In 2011, the diagnostic criteria for IgG4-related kidney disease (IgG4-RKD) were proposed by the IgG4-RKD working group of the Japanese Society of Nephrology (JSN) (Table 1) [5] and approved by the MHLW as organ-specific diagnostic criteria.

**Table 1:** Diagnostic criteria for IgG4-related kidney disease (IgG4-RKD) 2011

1. Presence of some kidney damage, as manifested by abnormal urinalysis or urine marker(s) or decreased kidney function with either elevated serum IgG level, hypocomplementemia, or elevated serum IgE level	
2. Abnormal renal radiologic findings:	
a. Multiple low-density lesions on enhanced computed tomography	
b. Diffuse kidney enlargement	
c. Hypovascular solitary mass in the kidney	
d. Hypertrophic lesion of renal pelvic wall without irregularity of the renal pelvic surface	
3. Elevated serum IgG4 level (IgG4 ≥ 135 mg/dl)	
4. Histologic findings in the kidney	
a. Dense lymphoplasmacytic infiltration with infiltrating IgG4-positive plasma cells > 10/high power field (HPF) and /or IgG4/IgG-positive plasma cells > 40%	
b. Characteristic fibrosis surrounding nests of lymphocytes and/or plasma cells	
5. Histologic findings in extra-renal organ(s):	
Dense lymphoplasmacytic infiltration with infiltrating IgG4-positive plasma cells > 10/HPF and/or IgG4/IgG-positive plasma cells > 40% in extra-renal organ(s)	
Definite:	
1 + 3 + 4a + 4b	
2 + 3 + 4a + 4b	
2 + 3 + 5	
1 + 3 + 4a + 5	
Probable:	
1 + 4a + 4b	
2 + 4a + 4b	
2 + 5	
3 + 4a + 4b	
Possible:	
1 + 3	
2 + 3	
1 + 4a	
2 + 4a	
Appendix:	
1. Clinically and histologically, the following diseases should be excluded: Wegener’s granulomatosis, Churg-Strauss syndrome, extramedullary plasmacytoma	
2. Radiologically, the following diseases should be excluded: malignant lymphoma, urinary tract carcinomas, renal infarction, and pyelonephritis (rarely, Wegener’s granulomatosis, sarcoidosis, and metastatic carcinoma)	

The aim of the present study was to determine the validity and potential problems of IgG4-RKD 2011 and devise modifications to finally develop a revised version for 2020.

## Validation of the diagnostic criteria for IgG4-related kidney disease 2011

### Patients and methods

Among patients diagnosed as having various forms of renal injury between April 2012 and May 2019 at the institutions affiliated to the IgG4-RKD working group, we retrospectively selected those for whom serum IgG4 values and/or data for immunohistological staining of IgG4 in renal biopsy samples were known, and then for study enrollment further selected those with sufficient clinical information, such as serological and radiological data and response to treatment. The IgG4-RKD working group consists of nephrologists, rheumatologists, and pathologists, all highly experienced in the diagnosis of IgG4-RKD. For each case, the physicians reported their degree of diagnostic confidence as “uncertain”, “slightly confident”, “confident” or “very confident” for IgG4-RKD or a mimicker, along with the clinical, serological, radiological, and histological data. Among those initially selected, only patients for whom investigators were “confident” or “very confident” about the diagnosis were included in this study, i.e., diagnoses made by physicians associated with the IgG4-RKD working group (experts) were regarded as the diagnostic gold standard for this study.

Thereafter, the included patients were classified as having “definite”, “probable”, “possible” or “unlikely” IgG4-RKD using the IgG4-RKD 2011 diagnostic criteria, and the results were compared with the expert opinions.

### Statistical analysis

The significance of differences between groups was determined using the paired Student’s *t* test, Mann–Whitney *U* test or Wilcoxon signed-rank test, and the significance of differences in frequencies was analyzed using Fisher’s exact probability test. Data are presented as means ± SD for continuous variables and as frequencies and percentages for categorical variables. A probability of *P* < 0.05 was considered to indicate statistical significance. Statistical analysis was performed using SPSS version 19 software (IBM SPSS, Chicago, IL, USA).

## Results

### General characteristics of mimickers and patients with true IgG4-RKD

Among the 116 collected patients, 105 for whom investigators were diagnostically “confident” or “very confident” were assessed. On the basis of expert opinion, 55 patients were diagnosed as having true IgG4-RKD and 50 as mimickers. Descriptions of patients in both groups are given in Table 2. The final clinicopathological diagnoses of the mimickers were anti-neutrophil cytoplasmic antibody (ANCA)-associated vasculitis [*n* = 8; microscopic polyangiitis (MPA) 5 patients, eosinophilic granulomatosis with polyangiitis (EGPA) 3 patients], idiopathic tubulointerstitial nephritis (TIN) (*n* = 5), drug-induced TIN (*n* = 5), nephrosclerosis (*n* = 4), Sj**ö**gren’s syndrome (*n* = 4), sarcoidosis (*n* = 3), multicentric Castleman’s disease (MCD) (*n* = 3), necrotizing glomerulonephritis without ANCA (*n* = 3), membranous nephropathy (n = 3), tubulointerstitial nephritis and uveitis (TINU) syndrome (*n* = 2), TIN associated with inflammatory bowel disease (*n* = 2), TIN with IgM-positive plasma cells (*n* = 2), TIN associated with infection (*n* = 2), IgA nephropathy (*n* = 1), diabetic nephropathy (*n* = 1), malignant lymphoma (*n* = 1) and antibody-mediated rejection after renal transplantation (*n* = 1).

**Table 2:** General characteristics and prevalence of individual items of IgG4-RKD diagnostic criteria with true IgG4-RKD and mimicker

	True IgG4-RKD (*n* = 55)	Mimicker (*n* = 50)	*P*
Age at diagnosis of IgG4-RKD, mean ± SD (years)	69.9 ± 9.4	56.7 ± 17.4	< 0.001
Male (%)	76.4	44	0.001
Allergy (%)	27.5	36.7	0.393
Serum IgG4 (mg/dl), mean ± SD	1028 ± 796	226 ± 261	< 0.001
Renal biopsy, performed (%)	92.7	100	0.120
Items of diagnostic criteria			
1. Presence of kidney damage with serological abnormalities^a^, *n*/total (%)	55/55 (100)	36/50 (72.0)	< 0.001
2. Abnormal renal radiologic findings^b^, *n*/total (%)	42/55 (76.4)	10/50 (20.0)	< 0.001
3. Elevated serum IgG4^c^, *n*/total (%)	54/55 (98.2)	18/50 (36.0)	< 0.001
4. Histological findings			
4a Dense IgG4 + PC^d^, *n*/total (%)	48/51 (94.1)	13/40 (32.5)	< 0.001
4b storiform fibrosis^e^, *n*/total (%)	28/51 (54.9)	3/50 (6)	< 0.001
5. Biopsy-proven extra-renal organ(s)^f^, *n*/total (%)	24/55 (43.6)	5/50 (10.0)	< 0.001

^a^Manifested by abnormal urinalysis or urine marker(s) and /or decreased kidney function with either elevated serum IgG level, hypocomplementemia, or elevated serum IgE level

^b^Multiple low-density lesions on enhanced CT, diffuse kidney enlargement, hypovascular solitary mass in the kidney, and hypertrophic lesion of the renal pelvic wall without irregularity of the renal pelvic surface

^c^Serum IgG4 level exceeding 135 mg/dl

^d^Dense lymphoplasmacytic infiltration with infiltrating IgG4-positive plasma cells > 10/high power field (HPF) and /or ratio of IgG4-positive plasma cells > 40%

^e^Characteristic “storiform” fibrosis surrounding nests of lymphocytes and/or plasma cells

^f^Extra-renal histology showing dense lymphoplasmacytic infiltration with infiltrating IgG4-positive plasma cells > 10/HPF and/or ratio of IgG4-positive plasma cells/IgG-positive plasma cells > 40%

Patients with true IgG4-RKD were significantly older with a male predominance and showed higher serum levels of IgG4 than mimickers. The frequency of allergy did not differ between the two groups. Most patients with true IgG4-RKD had an elevated serum IgG4 level and dense infiltration of IgG4-positive plasma cells in the renal tissue (98.2% and 94.2%, respectively). On the other hand, 18 of the 50 mimickers (36.0%) had an elevated serum IgG4 level (MCD 3, EGPA 3, MPA 2, TINU syndrome 2, and others 8). Dense infiltration of IgG4-positive plasma cells in the kidney was evident in 13 of 40 mimickers (32.5%; EGPA 3, MCD 2, MPA 2, drug-induced TIN 2, and others 4). Storiform fibrosis was evident in 28 of the 51 patients with true IgG4-RKD (54.9%) and 3 of the 50 mimickers (6.0%) (all 3 having EGPA) (Table 2).

### Performance of the IgG4-RKD 2011 diagnostic criteria

Among the 55 patients with true IgG4-RKD, 39 were classified as “definite”, 1 as “probable”, 15 as “possible” and none as “unlikely” based on the IgG4-RKD 2011 diagnostic criteria. Among the 50 mimickers, 5 were classified as “definite”, none as “probable”, 15 as “possible” and 30 as “unlikely” to have IgG4-RKD. Because cases classified as “possible” included any types of kidney disease with an elevated serum IgG4 level or IgG4-positive plasma cell infiltration in the renal tissue, many mimickers might have fallen into the category. In fact, only 15 of the 30 “possible” cases were true IgG4-RKD (diagnostic accuracy 50%). Conversely, no case was classified as “probable” among the mimickers. Therefore, we defined “definite” and “probable” cases based on the IgG4-RKD diagnostic criteria as “IgG4-RKD”, and “possible” and “unlikely” cases as “non-IgG4-RKD” in this study.

As a result, 40 of the 55 patients with true IgG4-RKD were classified as IgG4-RKD (39 definite and 1 probable): i.e., the sensitivity was 72.7% (95% confidence interval 59.8–82.7) and 45 of the 50 mimickers were classified as non-IgG4-RKD (15 possible and 30 unlikely): i.e., the specificity was 90.0% (95% confidence interval 78.6–95.7).

Table 3 shows the clinicopathological findings in the 15 patients with true IgG4-RKD who were classified as non-IgG4-RKD. Among them, IgG4-positive plasma cell-rich TIN was evident in 12 patients, but storiform fibrosis was absent. In 2 patients with TIN (nos. 10 and 12), IgG4-positively staining cells were not clearly enumerated because of the quality of the immunostaining, although storiform fibrosis was evident in 1 patient (no. 10). In 1 patient (no. 6), the renal histology was almost normal in the samples obtained, although sampling error was suspected. Thirteen of the 15 patients had extra-renal lesions. Although not all of these lesions were proven to be IgG4-associated histologically, 11 of the 13 patients had clinical or radiological findings suggestive of IgG4-RD, such as bilateral salivary gland swelling or retroperitoneal fibrosis. Table 4 shows the clinicopathological findings in the 5 mimickers who were classified as having IgG4-RKD (definite 5, probable 0). Their final diagnoses were EGPA (*n* = 3), MCD (*n* = 1) and malignant lymphoma (*n* = 1).

**Table 3:** True IgG4-RKD patients classified as non-IgG4-RKD according to the diagnostic criteria for IgG4-RKD 2011

	Renal radiologic findings	Serum IgG (mg/dl)	Serum IgG4 (mg/dl)	HC	Renal histology	IgG4 + PCs in renal interstitum (*n*/hpf)	Storiform fibrosis	Extra-renal lesions (without histology)
1		1964	170	−	TIN	> 50	−	
2		3190	860	+	TIN	> 50	−	Sa(bil)
3	A	3316	548	+	TIN + MN	20–49	−	Sa(bil),Ly,Lu, RPF
4	B	2328	378	+	TIN	> 50	−	
5	A	2244	503	−	TIN	> 50	−	RPF
6	A	1882	223	+	minor	0	−	La(bil), Sa(bil)
7	A	4891	1430	+	TIN	> 50	−	Sa(bil),Par(bil),Pa,Ly,Lu
8	A	2102	504	−	TIN + FSGS	20–49	−	Ly,RPF
9		2632	188	+	TIN	20–49	−	Lu
10		4457	1430	+	TIN + MN	Uninterpretable	+	La(bil),Sa(bil),Par(bil)Ly,Lu
11	B	5719	2800	−	TIN	10–19	−	Sa(uni)
12	A, B	2079	249	+	TIN	Uninterpretable	−	RPF
13	A	2400	709	+	TIN	20–49	−	La(bil), Sa(bil)
14		4140	634	+	TIN	20–49	−	Sa(bil), Ly
15	C	3178	528	+	TIN	10–19	−	Sa(bil), Par(bil)

A: multiple low-density lesions on enhanced CT, B: diffuse bilateral renal swelling, C: diffuse thickening of the renal pelvis wall with a smooth intra-luminal surface

*HC* hypocomplementemia, *IgG4 + PCs* IgG4-positive plasma cells, *TIN* tubulointerstitial nephritis, *MN* membranous nephropathy, *minor* minor abnormalities, *FSGS* focal segmental glomerulosclerosis, *bil* bilateral, *uni* unilateral, *Sa* sialadenitis, *Ly* lymphadenitis, *Lu* lung lesion, *RPF* retroperitoneal fibrosis, *La* dacryoadenitis, *Par* parotiditis

**Table 4:** Mimickers classified as IgG4-RKD according to the diagnostic criteria for IgG4-RKD 2011

	Renal radiologic findings	Serum IgG (mg/dl)	Serum IgG4 (mg/dl)	HC	Renal histology	IgG4 + PCs in renal interstitium (/hpf)	Storiform fibrosis	Extra-renal lesions	Final diagnosis
1		1995	645	−	TIN	20–49	−	sinusitis	EGPA(ANCA +)
2	A	2548	457	−	TIN + CrGN	> 50	+		EGPA(ANCA +)
3	A	2443	331	−	TIN + CrGN	20–49	+		EGPA(ANCA +)
4		4854	961	−	TIN	> 50		Ly	MCD
5	B	1992	595	−	TIN	10–19		Ly	Malignant lymphoma

A: multiple low-density lesions on enhanced CT, B: Diffuse bilateral renal swelling

*HC*; hypocomplementemia, *IgG4 + PCs* IgG4-positive plasma cells, *TIN* tubulointerstitial nephritis, *CrGN* crescentic glomerulonephritis, *Ly* lymphadenopathy, *EGPA* eosinophilic granulomatosis with polyangiitis, *MCD* multicentric Castleman’s disease

## Potential problems in the IgG4-RKD 2011 diagnostic criteria and development of the revised criteria

The results of the validation study and the comparison with CD for IgG4-RD highlighted the points below as potentially problematic. We developed some revised versions of the criteria and examined the sensitivity, specificity, positive likelihood ratio, and negative likelihood ratio for each revised version in the present cohort. On this basis, we selected the version showing the best performance (i.e. the one that most closely approximated our goal; positive and negative likelihood ratio > 10.0 and < 0.1, respectively) as the IgG4-RKD criteria for 2020.

### The number of IgG4-positive plasma cells and the ratio of IgG4/IgG-positive plasma cells

In the CD criteria for IgG4-RD 2011 [3], the pathology-related item: dense lymphoplasmacytic infiltration with (a) IgG4-positive plasma cells > 10/high power field (HPF) and (b) IgG4/IgG-positive plasma cells > 40%, is essential for diagnosis. However, in the IgG4-RKD 2011 diagnostic criteria, “(a) and/or (b)” is permitted for histological findings in the kidney. We validated this point using the present cohort. When we changed the term “(a) and/or (b)” to “(a) and (b)” (plan A), the sensitivity was decreased from 72.7% to 62.0%, while the specificity was unchanged at 90.0%. This was because IgG staining was uninterpretable due to technical problems in 3 patients and the ratio of IgG4/IgG-positive cells was < 40% in 4 patients with true IgG4-RKD.

In extra-renal organ(s), we changed the term “(a) and/or (b)” in the previous version to “(a) and (b)”, according to the other organ-specific classification criteria (the sensitivity and specificity were not altered by this change in the present cohort).

### Characteristic fibrosis surrounding nests of lymphocytes and/or plasma cells [so-called storiform fibrosis (or Bird’s eye)] in the renal pathology

In the present cohort, “storiform fibrosis” in the renal pathology was evident in 54.9% of patients with true IgG4-RKD, which was a lower frequency than expected (Table 2). Fourteen of 15 patients with true IgG4-RKD who were classified as having non-IgG4-RKD lacked such storiform fibrosis (Table 3). In the present cohort, we tested the performance of the modified version (plan B) in which the item “storiform fibrosis” was deleted from among histologic findings in the kidney. As a result, the sensitivity increased from 72.7 to 94.5%, but the specificity was markedly decreased from 90.0 to 76.0%.

### Extra-renal organ involvement(s)

In the IgG4-RKD 2011 diagnostic criteria, histological evidence in the affected organ(s) was mandatory for the diagnosis of the extra-renal lesion(s). However, some characteristic clinical or radiological findings have been shown to be quite useful for the diagnosis of IgG4-RD, as is the case for histological features. For example, the clinical finding “two or more sets of the bilateral lacrimal, parotid, sublingual or submandibular glands swellings” or the radiological finding “diffuse pancreas enlargement and capsule-like rim with decreased enhancement” are considered to be highly suggestive for IgG4-RD, even in the absence of histology [6]. Table 5 shows extra-renal organ involvement(s) in patients with true IgG4-RKD and mimickers. The results show that clinical findings of bilateral lacrimal, parotid or submandibular gland swellings and radiological findings compatible with type 1 autoimmune pancreatitis or retroperitoneal fibrosis were frequently evident in true IgG4-RKD but rare in mimickers. We, therefore, added the item “imaging or clinical findings compatible with extra-renal organ(s)” as an extra-renal organ(s) item. We prepared 4 patterns (plans C-1 to C-4) for this item and tested the performance of each in the present cohort.

**Table 5:** Extra-renal organ involvement(s) in true IgG4-RKD and mimicker

	True IgG4-RKD (*n* = 55)	Mimicker (*n* = 50)
Ocular lesion(s), *n*	0	1 (uveitis)
Neuritis, *n*	1	0
Sinusitis, *n*	2	2
Skin lesion(s), *n*	1	4
Lacrimal gland swelling (unilateral), *n*	1	0
Lacrimal gland swelling (bilateral), *n*	15	0
Parotid gland swelling (unilateral), *n*	0	1
Parotid gland swelling (bilateral), *n*	8	0
Salivary gland swelling (unilateral), *n*	2	0
Salivary gland swelling (bilateral), *n*	27	0
Lymphadenopathy, *n*	21	7
Lung lesion(s), *n*	16	4
Type 1 autoimmune pancreatitis, *n*	13	0
Retroperitoneal fibrosis, *n*	9	1

C-1; (1) bilateral lacrimal gland swelling or (2) bilateral submandibular or parotid gland swelling or (3) imaging findings compatible with type 1 autoimmune pancreatitis, or 4) imaging features of retroperitoneal fibrosis.

C-2; (1) and (2), or (3), or (4).

C-3; (1) or (2), or (3).

C-4; (1) and (2), or (3).

The sensitivity and specificity of the modified classification criteria for IgG4-RKD using each pattern were: (C-1) 90.9% and 90.0%, (C-2) 87.3% and 90.0%, (C-3) 85.5% and 90.0% and (C-4) 80.0% and 90.0%, respectively.

Table 6 shows the sensitivity, specificity, positive likelihood ratio and negative likelihood ratio of the IgG4-RKD 2011 version, plans A, B, and C1-C4. Among them, plan C-1 showed the highest performance (positive likelihood ratio 9.09 and negative likelihood ratio 0.10), and therefore we selected it as the IgG4-RKD criteria for 2020.

**Table 6:** Performance of the original version and each revised version

	Sensitivity (%)	Specificity (%)	Positive likelihood ratio	Negative likelihood ratio
2011 criteria	72.7	90.0	7.27	0.30
Plan A	62.0	90.0	6.20	0.42
Plan B	94.5	76.0	3.94	0.07
Plan C-1	90.0	90.0	9.09	0.10
Plan C-2	87.3	90.0	8.73	0.14
Plan C-3	85.5	90.0	8.55	0.16
Plan C-4	80.0	90.0	8.00	0.22

Plan A: Change the pathology-related item: dense lymphoplasmacytic infiltration with (a) IgG4-positive plasma cells > 10/high power field (HPF) and/or (b) IgG4/IgG-positive plasma cells > 40% to “(a) and (b)”

Plan B: Deletion of the item “storiform fibrosis” from among histologic findings in the kidney

Plan C: Addition the item “imaging or clinical findings compatible with extra-renal organ(s)” as an extra-renal organ(s) item

Plan C-1: (1) bilateral lacrimal gland swelling or (2) bilateral submandibular or parotid gland swelling, or (3) imaging findings compatible with type 1 autoimmune pancreatitis, or (4) imaging features of retroperitoneal fibrosis

Plan C-2: (1) and (2), or (3), or (4)

Plan C-3: (1) or (2), or (3)

Plan C-4: (1) and (2), or (3)

### Mimickers

An elevated serum IgG4 level and infiltration of abundant IgG4-positive plasma cells have been considered features of ANCA-associated vasculitis, including MPA, MCD, and malignant lymphoma [7–9], and indeed these findings were confirmed in the present cohort. Here, we changed the term “GPA and EGPA” in the previous version to “ANCA-associated vasculitis” and added MCD and malignant lymphoma as diseases required for differential diagnosis, as detailed in the Appendix section of the revised version.

## Proposal of diagnostic criteria and a diagnostic algorithm for IgG4-RKD 2020

The revised version of the diagnostic criteria for IgG4-RKD 2011 (i.e. the diagnostic criteria for IgG4-RKD 2020) are shown in Table 7. Although 3 + 4a + 4b” was “probable” in the original version (Table 1), we deleted it from “probable” in the revised version because 1 or 2 is essential as an entry criterion in clinical practice.

**Table 7:** Diagnostic criteria for IgG4-related kidney disease (IgG4-RKD) 2020

1. Presence of some kidney damage, as manifested by abnormal urinalysis or urine marker(s) or decreased kidney function with either elevated serum IgG level, hypocomplementemia, or elevated serum IgE level	
2. Abnormal renal radiologic findings:	
a. Multiple low-density lesions on enhanced computed tomography	
b. Diffuse kidney enlargement	
c. Hypovascular solitary mass in the kidney	
d. Hypertrophic lesion of the renal pelvic wall without irregularity of the renal pelvic surface	
3. Elevated serum IgG4 level (IgG4 ≥ 135 mg/dl)	
4. Histologic findings in the kidney	
a. Dense lymphoplasmacytic infiltration with infiltrating IgG4-positive plasma cells > 10/high power field (HPF) and /or IgG4/IgG-positive plasma cells > 40%	
b. Characteristic fibrosis surrounding nests of lymphocytes and/or plasma cells	
5. Extra-renal organ(s):	
a. Dense lymphoplasmacytic infiltration with infiltrating IgG4-positive plasma cells > 10/HPF and IgG4/IgG-positive plasma cells > 40% in extra-renal organ(s)	
b. Imaging or clinical findings in extra-renal organ(s): existence of one of the following items:	
(1) Bilateral lacrimal gland swelling	
(2) Bilateral submandibular or parotid gland swelling	
(3) Imaging findings compatible with type 1 autoimmune pancreatitis	
(4) Imaging features of retroperitoneal fibrosis	
Definite:	
1 + 3 + 4a + 4b	
2 + 3 + 4a + 4b	
2 + 3 + 5a	
1 + 3 + 4a + 5a or 5b	
2 + 3 + 4a + 5b	
Probable:	
1 + 4a + 4b	
2 + 4a + 4b	
2 + 5a	
2 + 3 + 5b	
Possible:	
1 + 3	
2 + 3	
1 + 4a	
2 + 4a	
2 + 5b	
Appendix	
1. Clinically and histologically, exclusion of the following diseases should be considered: ANCA-associated vasculitis, multicentric Castleman’s disease, malignant lymphoma, and extramedullary plasmacytoma	
2. Radiologically, exclusion of the following diseases should be considered: malignant lymphoma, urinary tract carcinoma, renal infarction and pyelonephritis (rarely, granulomatosis with polyangiitis, sarcoidosis and metastatic carcinoma)	

Using the revised criteria, 48 of 55 true IgG4-RKD cases were classified as “definite”, 2 as “probable”, and 5 as “possible”, i.e. the sensitivity improved to 90.9%. The performance among the mimickers was unchanged from the previous version (5 were classified as “definite”, none as “probable”, 15 as “possible” and 30 as “unlikely”), i.e. the specificity remained at 90.0%.

**Fig. 1:**
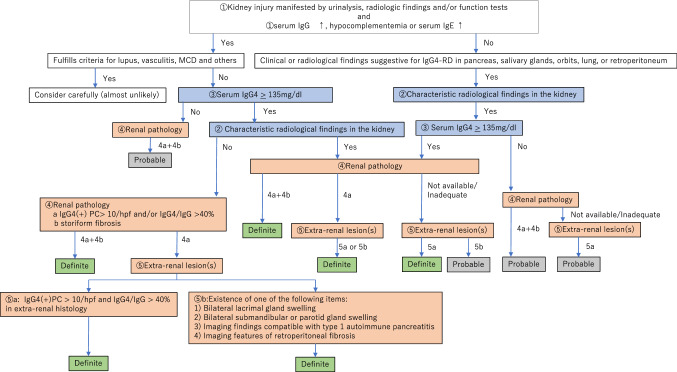
The diagnostic algorithm for IgG4-RKD 2020

**Fig. 2:**
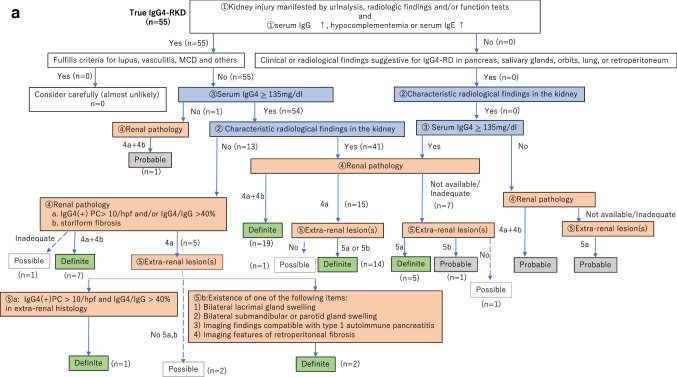

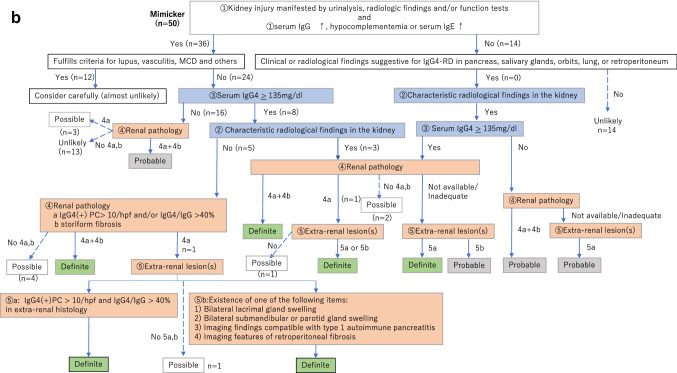
The performance of the diagnostic algorithm for IgG4-RKD among the cases of true IgG4-RKD (**a**) and mimickers (**b**)

The revised version of the diagnostic algorithm for IgG4-RKD (i.e. the diagnostic algorithm for IgG4-RKD 2020) is shown in Fig. 1. Because this algorithm is used to select definite and probable IgG4-RKD cases in clinical practice, any case fulfilling the criteria for known diagnoses such as lupus, Sjögren’s syndrome, vasculitis, MCD or malignant lymphoma comes under the arm “consider carefully (almost unlikely)”. Especially, as an elevated serum IgG4 level and infiltration of abundant IgG4-positive plasma cells may be evident in ANCA-associated vasculitis, MCD, and malignant lymphoma [7–9], such cases need to be considered very carefully, although a few overlapping cases have been reported [10]. Figure 2 shows the performance of the diagnostic algorithm for IgG4-RKD among the cases of true IgG4-RKD and mimickers in the present cohort. Using this algorithm, 90.9% of true IgG4-RKD cases were diagnosed as definite or probable IgG4-RKD (Fig. 2a), while none of the mimickers were diagnosed as IgG4-RKD (Fig. 2b).

## Discussion

The kidney is one of the organs most frequently affected in IgG4-RD [11]. Although IgG4-related TIN is the most dominant feature [12], some glomerular lesions concurrent with IgG4-related TIN have also been reported and several radiologically identified kidney lesions including pelvic lesions have been shown to be characteristic of IgG4-RD and useful for its diagnosis, in the setting of other forms of organ involvement that have been confirmed histopathologically. Therefore, the term “IgG4-RKD” has been proposed as a comprehensive term for the renal lesions associated with IgG4-RD, including IgG4-related TIN, glomerular lesions concurrent with IgG4-related TIN, and IgG4-related pyelitis, and the diagnostic criteria for IgG4-RKD were published in 2011 by the IgG4-RKD working group of the JSN [5]. The present study represents the first attempt to validate these diagnostic criteria among Japanese kidney patients collected retrospectively after 2011. Here we found that the sensitivity and specificity of the IgG4-RKD 2011 diagnostic criteria were 72.7% and 90.0%, respectively, suggesting that they are highly specific but have relatively low sensitivity. An additional potential problem was the lack of unity in the CD criteria for IgG4-RD 2011 or organ-specific criteria except for kidney disease, in terms of the number of IgG4-positive plasma cells and the ratio of IgG4/IgG-positive plasma cells.

To test some revised versions, we prepared several patterns (plans A, B and C1-4) and examined the sensitivity and specificity of each. With regard to infiltrating IgG4-positive cells in the renal histology, we confirmed that “(a) IgG4-positive plasma cells > 10/hpf and/or (b) IgG4/IgG-positive plasma cells > 40%” was more appropriate than “(a) and (b)” for diagnosis of IgG4-TIN, as had been previously suggested by Kawano, et al. [13]. In addition, we confirmed “storiform fibrosis” to be an important item for the specificity of IgG4-RKD diagnosis, although the frequency was lower than that in previous studies [14].

With regard to “imaging or clinical findings compatible with extra-renal organ(s)”, we selected plan C-1 [(1) bilateral lacrimal gland swelling or (2) bilateral submandibular or parotid gland swelling or (3) imaging findings compatible with type 1 autoimmune pancreatitis, or (4) imaging features of retroperitoneal fibrosis] based on the results for the positive likelihood ratio and negative likelihood ratio. However, it should always be borne in mind that such imaging or clinical features might be evident in mimickers. In particular, retroperitoneal fibrosis as an imaging feature has been demonstrated in ANCA-associated vasculitis or malignant lymphoma [9, 15, 16]. In fact, the present cohort included one patient with MPA who showed imaging features of retroperitoneal fibrosis and IgG4-positive cell-rich TIN, although the level of serum IgG4 was not evaluated. If a high serum IgG4 level had been evident, this patient would have been misclassified as “IgG4-RKD” according to the revised version.

In summary, we have validated the diagnostic criteria for IgG4-RKD 2011 and proposed a revised version (the diagnostic criteria for IgG4-RKD 2020). Use of the revised criteria improved the sensitivity to 90.9%, while the specificity remained at 90.0%, being unchanged from the previous version. Prospective studies to validate the revised version in a separate cohort will be necessary.
